# Green and Integrated Wearable Electrochemical Sensor for Chloride Detection in Sweat

**DOI:** 10.3390/s22218223

**Published:** 2022-10-27

**Authors:** Francesco Lopresti, Bernardo Patella, Vito Divita, Claudio Zanca, Luigi Botta, Norbert Radacsi, Alan O’Riordan, Giuseppe Aiello, Maïwenn Kersaudy-Kerhoas, Rosalinda Inguanta, Vincenzo La Carrubba

**Affiliations:** 1 Department of Engineering, University of Palermo, RU INSTM of Palermo, Viale delle Scienze, 90128 Palermo, Italy; 2 School of Engineering, Institute for Materials and Processes, The University of Edinburgh, King’s Buildings, Robert Stevenson Road, Edinburgh EH9 3FB, UK; 3 Nanotechnology Group, Tyndall National Institute, University College Cork, T12R5CP Cork, Ireland; 4 Institute of Biological Chemistry, Biophysics and Bioengineering, Heriot-Watt University, Edinburgh EH14 4AS, UK

**Keywords:** electrochemical sensors, wearable sensor, chloride detection, electrolyte assisted electrospinning, environmental-friendly, laser cutting

## Abstract

Wearable sensors for sweat biomarkers can provide facile analyte capability and monitoring for several diseases. In this work, a green wearable sensor for sweat absorption and chloride sensing is presented. In order to produce a sustainable device, polylactic acid (PLA) was used for both the substrate and the sweat absorption pad fabrication. The sensor material for chloride detection consisted of silver-based reference, working, and counter electrodes obtained from upcycled compact discs. The PLA substrates were prepared by thermal bonding of PLA sheets obtained via a flat die extruder, prototyped in single functional layers via CO_2_ laser cutting, and bonded via hot-press. The effect of cold plasma treatment on the transparency and bonding strength of PLA sheets was investigated. The PLA membrane, to act as a sweat absorption pad, was directly deposited onto the membrane holder layer by means of an electrolyte-assisted electrospinning technique. The membrane adhesion capacity was investigated by indentation tests in both dry and wet modes. The integrated device made of PLA and silver-based electrodes was used to quantify chloride ions. The calibration tests revealed that the proposed sensor platform could quantify chloride ions in a sensitive and reproducible way. The chloride ions were also quantified in a real sweat sample collected from a healthy volunteer. Therefore, we demonstrated the feasibility of a green and integrated sweat sensor that can be applied directly on human skin to quantify chloride ions.

## 1. Introduction

The demand for wearable sensors (WS) for sweat biomarkers detection is rapidly increasing since they can easily provide analyte access to a range of analytes and permit monitoring for several diseases in a non-invasive manner [1,2,3]. Ideally, materials and manufacturing methods for the fabrication of WS should ensure high sensitivity, reproducibility, selectivity, and biocompatibility of both sensors and platforms [1,4]. Furthermore, the entire device must be mechanically adequate for dynamic use on human skin [5,6]. In addition to these requirements, the environmental sustainability of new point-of-care devices, especially those for single-use applications, should be considered [7].

There are different types of WS, including colorimetric, mechanical, optical, and electrochemical [8,9,10]. Among these, electrochemical sensors are particularly attractive because they can operate with a small sample volume and provide a quick, fast, remote, and continuous response [11,12,13]. They can be used for the detection of a wide range of complex analytes also of a complex nature, such as proteins, neurotransmitters, and hormones [11,14,15,16,17,18].

For a good electrochemical WS for sweat analysis, it is essential to optimize three different parts: the substrate for the device construction, the absorbent material for in situ sweat sampling, and the active material of the working electrode for the electrochemical detection of the target analyte [9,19,20]. A key requirement is that the substrate obviously must possess numerous qualities such as flexibility and mechanical stability [1,21]. 

The most popular materials investigated and adopted as a substrate for WS fabrication include poly(dimethylsiloxane) (PDMS) [22,23,24,25], polyethylene terephthalate (PET) [26,27,28,29], and poly(methyl methacrylate) (PMMA) [30]. However, major concerns associated with the non-renewability and non-biodegradability of traditional thermoplastics derived from fossil fuels are increasing the demand for more eco-sustainable alternatives [31]. To this end, in this work, the attention was focused on polylactic acid (PLA). PLA can be considered a suitable candidate for substrate fabrication due to its biocompatibility, inertness to small molecules, optical qualities, and suitability for laser cutting [23,32]. Furthermore, PLA is a more environmentally sustainable choice compared to other fossil-based thermoplastic polymers due to its composability and renewable resource derivation [33].

We have demonstrated a number of PLA-based microdevices; however, efficient and reliable bonding remains a challenge [23,32]. Among the bonding approaches for thermoplastic polymers, the simplest is thermal bonding, although high temperatures can damage micropatterns on polymeric sheets [34,35]. Surface treatments can be used to improve the bonding strength of thermoplastic polymers at lower processing temperatures [36,37,38]. Among them, plasma treatment is a solvent-free and sustainable [39] surface modification approach used for improving bonding strength in microfluidic chips fabricated with different thermoplastic polymers [36,40,41,42,43,44,45,46,47,48]. In this work, for the first time, air-plasma modification was systematically investigated to improve the bonding strength of PLA sheets for miniaturized WS manufacturing.

Another key requirement for WS development is that the active material of the working electrode is highly selective towards the target analyte. In this work, chloride ions were selected as a target analyte for detection in sweat. This analyte is of great importance for human health. Anomalous concentrations are associated with various diseases such as metabolic acidosis and alkalosis coagulation dysfunction, kidney and liver diseases, heart failure, high blood pressure, diabetes, Addison’s disease, and cystic fibrosis [49,50,51,52]. In addition, during physical activity, the chloride concentration in sweat decreases, and this is related to the onset of muscle cramps [53,54]. Nowadays, chloride ions are quantified using different lab-based techniques, which do not allow for the on-body analysis of sweat [55]. In particular, the most common analytical methods are: inductively coupled plasma mass spectrometry [56], fluorescent chemosensor [57], ion chromatography [58], and turbidimetric methods [59].

For wearable applications, electrochemical sensors are perfect candidates to guarantee, with very high sensitivity, selectivity, and on-body analysis of sweat with the use of a small potentiostat [60,61,62,63,64,65]. Recently [66], we have studied the possibility of building electrochemical sensors for chloride ions based on the electrochemical silver-to-silver chloride reaction. In that work, silver-based sensors were used that were developed using upcycled compact discs (CDs) by laser cutting. The sensor platform consisted of silver-based working, counter, and reference electrodes. The electrode manufacturing was very fast (<1 min/sensor), cheap (<0.5 euro/sensor) and green as it started from an obsolete material such as CDs. In our previous work, we demonstrated that the electrode allowed the detection of Cl^−^ in a wide linear range, from 0.05 to 50 mM, with very high sensitivity [66,67], and could quantify chloride ions in different water-based liquids, including sweat.

However, the sensor tested in [66] was unable to be directly applied to human skin for the quantification of chloride ions due to the absence of an absorbent material that is in contact with the skin and acts as in-situ sweat sampling [2]. Here, an electrospun and free-standing membrane made of PLA was integrated into the WS as absorbent materials for direct sweat sampling.

In this work, we propose, for the first time, a WS for sweat absorption and electrochemical analysis of chloride ions based on PLA (used both for the substrate and sorbent membrane) and electrochemical sensors developed from CDs [66]. PLA sheets of different thicknesses were obtained via a flat die-headed extruder and prototyped in single functional layers via CO_2_ laser cutting. The surface of the layers was etched by air plasma at different processing powers and times and finally assembled using a layer-by-layer based technique [23,34]. The plasma treatment parameters were optimized by monitoring the bonding strength via mechanical and burst tests and the optical transparency via UV-Vis analysis. The electrospun PLA membrane, used as a sweat absorption pad, was directly deposited on the membrane holder layer by means of the electrolyte-assisted electrospinning technique, thus avoiding misalignment and damage due to cutting and user-handling [68]. The efficacy of the membrane adhesion to the chip using the different processing parameters was investigated by indentation tests in dry and wet conditions. The silver-based electrodes (pseudo-reference, working, and counter electrodes) obtained from CDs were obtained by laser cutting and assembled into a microfluidic chip together with the PLA sheets and electrospun membrane. In this way, it was possible to obtain a complete, flexible, and functional device that is easily adaptable to the human body. The sensor was used for the quantification of chloride ions in sweat, but in general, it can be used for all those analytes for which silver has high sensitivity and selectivity. The device demonstrated excellent mechanical properties and very good features for chloride ions detection. It is also validated using real sweat and achieved results comparable with conventional techniques.

## 2. Materials and Methods

### 2.1. Materials and Device Fabrication

PLA (2003D, extrusion grade) was supplied by NatureWorks^®^ (Natureworks LLC, Minnetonka, MN, USA) while Acetone (Ac), sodium nitrate, sodium chloride, and chloroform (TCM) were purchased from Sigma Aldrich, Munich, Germany. All the solutions were made using deionized water. 

To prepare the flat PLA sheets, an extruder (Plastograph EC-Plus, 19/25D, Brabender GmbH & Co. KG, Duisburg, Germany) equipped with a flat die head and with a calender was used. The chosen temperature profile was: T_1_ = 180 °C; T_2_ = 190 °C; T_3_ = 200 °C; T_die_ = 200 °C. The extruder screw speed was 18 rpm, while the calender speed was set in the range 0.5–1 rpm in order to fabricate sheets 0.5–0.2 mm thick.

The design of the different layers composing the final device was performed using CAD software (Fusion360, v2019, Autodesk Inc., Mill Valley, CA, USA). The PLA layers were cut using a CO_2_ laser cutter (Maitech, 50 W, Varese, Italy). Laser power, speed, and frequency were chosen as input parameters to obtain the best PLA laser cutting conditions for each layer thickness according to [32]. For PLA sheets 0.2 mm thick, the pass-through operation was performed by setting the laser speed equal to 20 mm/s and power equal to 40%. The cut at the same operative conditions was repeated twice for PLA sheets 0.5 mm thick. The engraving operation was performed by setting the laser speed equal to 160 mm/s and power equal to 30%. For the deeper engraved region, the process was repeated twice. The cut layers were cleaned in pure ethanol and then functionalized by a cold plasma reactor (Tucano, Gambetti kenologia SRL, Binasco, Italy) equipped with a polarized anode (RF = 13.56 MHz). To evaluate the influence of the plasma treatment parameters on the bonding efficiency of the PLA layers, the treatment was carried out at two different power levels (100 W and 150 W) and three different times (120, 180, and 240 s). Both surfaces of the PLA layers were treated with plasma. In addition, the electrospun membrane surfaces were plasma treated using the same equipment set at 50 W for 30 s. 

The Ag films were removed from the CDs by means of a tape and cut by setting the laser speed equal to 100 mm/s and power equal to 10% to minimize damage to the thin silver film. The silver film was cut into three different parts to obtain the working, counter, and reference electrode. 

PLA layers were bonded together (within 3 h following plasma treatment) by sandwiching them between custom-made plates equipped with pins for alignment of the individual layers. The sandwiched layers were kept in conformal contact at 50 °C for 10 min in a Carver laboratory press at 30 bar, and then rapidly cooled down to room temperature [23].

The PLA-based sweat absorption membranes were prepared via electrolyte-assisted electrospinning. Specifically, PLA was solubilized in a mixture at 10 wt% of chloroform and acetone (2:1 vol) under continuous magnetic stirring overnight. Electrospinning equipment (NF-103, MECC CO., Ltd., Fukuoka, Japan) was used to prepare PLA microfibrous membranes. A 5-mL syringe equipped with a 19-gauge stainless steel needle was used as a reservoir for the polymeric solution. The electrospinning process was then carried out by setting the following parameters: flow rate 1 mL/h, needle-collector distance 13 cm, voltage 15 kV, temperature 25 °C, relative humidity 40%, needle x-axes stage course 7 cm, needle x-axes speed 1 mm/min. An electrolytic solution (CaCl_2_ 10 wt% in water) was used as a static collector for precise fiber deposition in the membrane holder layer of the device. For this purpose, the electrolytic solution was directly deposited in the membrane holder layer. The processing time was set at 5 min to obtain two square-shaped membranes approximately 50 μm thick.

### 2.2. Characterization

The transparency of the materials before and after bonding was tested by analyzing the transmittance of the light through the samples in the visible region using a UV-vis spectrophotometer (model UVPC 2401, Shimadzu Italia s.r.l., Milan, Italy). 

Static water contact angle (WCA) measurements were carried out with an FTA 1000 (First Ten Ångstroms, Portsmouth, UK) by using distilled water as fluid. The test was performed on PLA sheets and on PLA electrospun membranes before and after plasma treatment. Different water drop images (7 spots of each sample) were taken at a time of 10 s, and the average value of WCA was calculated.

Shear stress testing was performed by adopting the single lap joint method with a universal testing machine (UTM, model 3367, Instron, High Wycombe, UK). The test speed was set at 0.05 mm/s until delamination of the samples was observed. The rectangular specimens (100 × 13.3 mm) were laser cut from 0.5 mm thick sheets and overlapped for a length of 30 mm (overlapped area 4 cm^2^). An additional layer was added to the double-sided sample on the gripping side to ensure alignment during the bonding and shear stress test. At least 5 specimens were used for each set of plasma parameters.

Chips featuring a 0.5 mm wide channel leading to a dead end 4 mm diameter round chamber were prepared by sandwiching a 0.2 mm thick layer between two 0.5 mm thick PLA layers. One channel also served for the fluidic connection ensured through flangeless ferrules, tubing, and connectors, tightened to the chip by a custom 3D printed screw/nut system. A syringe pump (Darwin Microfluidics, Paris, France) equipped with a 5-mL syringe was used to push deionized water in the dead-end channel at 0.5 mL/min. A LabSmith pressure sensor (uPS-series, model: uPS1800-T116-10, Mengel Engineering, Virum, Denmark) was used to monitor the pressure changes as a function of time. The highest pressure reached before chip failure was considered the burst limit of the specimens. At least three specimens were tested for each plasma condition.

The morphology of the PLA cut samples was analyzed via Dino-Lite digital microscope (Dino-Lite, model AM4115T-CFVW, AnMo Electronics Corp., Hsinchu, Taiwan). To evaluate the morphology of the electrospun membranes, a scanning electron microscopy (Phenom ProX, Phenom-World, Eindhoven, The Netherlands) at an accelerated voltage of 10 kV was used. The membrane SEM images were taken before and after plasma treatment and after the integration within the PLA layers via thermal bonding. Five frames for each sample were analyzed with a dedicated plugin software (DiameterJ) to estimate the mean fiber diameter and mean pore dimension [69].

Indentation experiments were performed on the bonded membranes in dry and wet conditions using the Instron UTM equipped with a spherical-tip indenter (3 mm in diameter). The samples are centered below the indenter tip to create a nearly frictionless contact between the spherical indenter probe and the membrane. The indenter tip was displaced at the rate of 1 mm/min [70].

The water uptake capacity of the electrospun PLA mats (*WU_PLA_*) was evaluated by gravimetric analysis (using a Sartorius AX224 with a resolution of ±0.1 mg) as the difference between the weight of the membrane filled with water (*W_wet_*) and the weight before water absorption (*W_dry_*) according to Equation (1)

(1) $$WU_{PLA}\left(\%\right)=\frac{W_{wet}-W_{dry}}{W_{dry}}\times 100$$

A helium pycnometer (Pycnomatic ATC, Porotec GmbH, Thermo Electron Corporation, Hofheim/ts., FRG) was used to measure the bulk density of PLA membranes. For each sample, ten measurements were carried out, and the average value was reported (standard deviations lower than 0.01 g/cm^3^).

The membrane porosity was evaluated according to the following Equation (2):

(2) $$Porosity\;\left(\%\right)=\left(1-\frac{\rho_{membrane}}{\rho_{matrix}}\right)\times 100$$

where *ρ_membrane_* is the apparent density of the membrane while *ρ_matrix_* is the bulk density of the mats, also evaluated via helium pycnometer.

### 2.3. Sensor Performance 

In our previous work [66], we have fully characterized and tested the Ag-based sensors obtained from CDs for chloride ions detection. The aim of this work is to show that these Ag-based sensors may also work in a complete device equipped with an absorbing membrane that puts the skin in contact with the electrochemically active part of the sensor, ensuring electrolyte contact. For this purpose, the three parts (working, reference and counter) of the sensor have been drawn on the upper surface of the CD, by means of a laser cutter. The CD surface layer consists of a thin polymeric film that protects the Ag layer. By suitably adjusting the laser power, it is possible to completely ablate the separation areas between the three parts of the sensor in order to avoid short circuits. After ablation, the complete sensor is peeled from the polycarbonate substrate of the CD and transferred to the PLA sheet in order to assemble the complete device. Thus, the fabricated devices were tested with and without the PLA electrospun membrane to verify its influence on the sensor performance. Sensors were tested using sodium nitrate 0.1 M as a blank solution. In this blank, different sodium chloride-concentrated solutions were added. Linear scan voltammetry, with a scan rate of 10 mV/s from −0.2 to 0.8 V, was used as an electrochemical technique [66]. The peak current density value was used as sensor output to build the calibration line. A portable potentiostat (EmStat3.0) was used to simulate the application of the proposed sensor directly on human skin. Each concentration was tested with a new and fresh device, and for each test, at least three devices were used. The sensor selectivity was evaluated against glucose, lactic acid, phosphate, sulphate and potassium ions. These species were selected as they can be found in the sweet. The interferents were added at a concentration of 10 mM that is very high in comparison to the normal concentration present in human sweat. This condition was chosen in order to simulate the worst possible case. The sensor was further validated using a real sweat sample collected from a healthy volunteer, and the results were compared with the chloride concentration measured by the chemical titration approach. In particular, the standard Mohr’s method was used [71].

## 3. Results and Discussion

### 3.1. Influence of Plasma Treatment on Transparency and Wettability of PLA Sheets

Flat-die-headed extrusion is a well-established processing technique for the continuous fabrication of thermoplastic polymer sheets with different thicknesses. The use of melt-based processing can be considered sustainable from an economic and environmental point of view since it allows for avoiding organic solvents, which are expensive and potentially dangerous for the environment.

The processing parameters related to the final thickness of the extruded sheet are mainly the extrusion screw speed and the calendar speed. In this work, the screw speed was maintained at 18 rpm while the thickness was easily tuned by the calendar speed. More in detail, PLA sheets with a thickness ranging from 0.2 to 0.5 mm were obtained by changing the calendar speed from 1 rpm to 0.5 rpm. The possibility of easy control of the sheet thickness is a key point since it strongly affects the final properties of the functional layer in the assembled chip device. 

**Figure 1:**
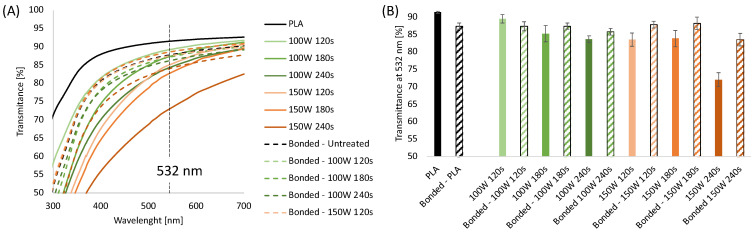
(**A**) UV-Vis spectra of extruded PLA sheets 0.2 mm thick with and without plasma treatments, before and after bonding; (**B**) Transmittance at 532 nm of 0.2 mm thick extruded PLA sheets with and without plasma treatments, before and after bonding.

The process allowed flat, smooth, and transparent samples to be obtained as the melt was rapidly cooled by air when it left the die. The transparency of the sheets was measured by UV-Vis transmittance. In Figure 1A, the UV-Vis spectra for 0.2 mm thick PLA sheets 0 with and without plasma treatments were reported. Air plasma treatment was carried out at 100 W and 150 W at different times (120 s, 180 s, and 240 s). UV-Vis spectra were also collected before and after bonding.

The transmittance values obtained at 532 nm are displayed in Figure 1B. The results show that the untreated PLA sheets showed a transparency of 92%, which decreased to 87% after the thermal bonding process due to an increase in the sample thickness. UV-Vis analysis conducted on plasma-etched samples showed it is possible to observe that the transparency of the sheets decreases upon increasing the etching time and power. Specifically, the transmittance of the sheets treated at 100 W for 120, 180, and 240 s was 89.5%, 85%, and 83%, respectively, while the transmittance of the PLA substrates treated at 150 W was 84% for 120 and 180 s, decreased down to 72% for the highest treatment time. Interestingly, after bonding, the plasma-treated samples presented higher transparency when compared to the corresponding unbonded samples, except for sheets treated at 100 W for 120 s. The transparency of the bonded plasma-treated layers is comparable to that of the bonded PLA samples (in the range 85–88%) independent of the plasma etching condition except for 150 W 240 s that showed a significantly reduced value of transmittance at 532 nm, down to 72%. These results can be likely explained by considering that the transparency of a polymeric sheet depends on the roughness of the sample. It is well known that plasma treatment induces granular-like surface microstructure on PLA, making it rougher than the untreated samples [37,72]. After bonding, the surficial effects of the plasma treatment were less evident, probably due to the contact between the two PLA layers each other and the sample holder.

**Figure 2:**
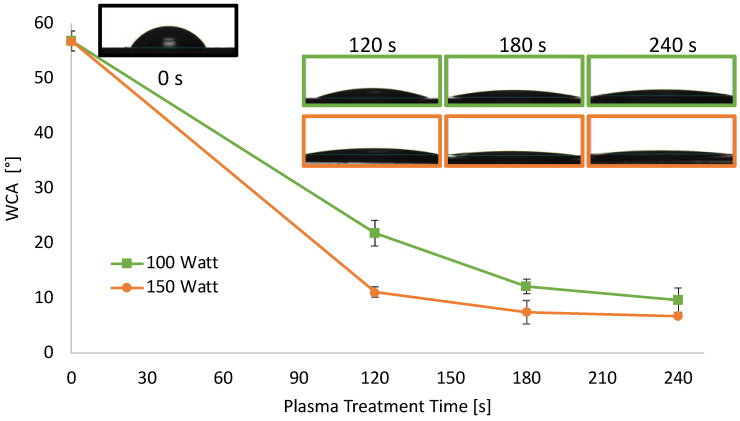
WCA of extruded PLA sheets 0.2 mm thick as a function of plasma treatment power and time.

In addition to the changes in transparency, also the wettability of the sheets underwent modifications after air plasma treatment. As reported in Figure 2, hydrophilicity changes were evaluated by measuring the WCA on the surface PLA sheets before and after air-plasma treatment. 

The WCA of PLA sheets decreased upon increasing the etching power and time, reaching a plateau after 180 s. Specifically, the WCA value of neat PLA sheets was 57° and, when treated at 100 W, decreased almost linearly down to 12° for a treatment time of 180 s, remaining almost constant above that. When air plasma power was set at 150 W, the WCA decrease was more dramatic, in the 120 s of treatment it reached 11°. WCA of PLA treated at 150 W was further decreased around 7°, after 180 and 240 s of treatment.

This hydrophilicity increase on the PLA surface was expected since air-oxygen plasma can create oxygenated moieties on the PLA surface, and, secondarily, surface polymeric chain degradation occurs. This test proved the effective surface functionalization of the PLA sheets [73].

### 3.2. Mechanical Tests on Bonded PLA Sheets

**Figure 3:**
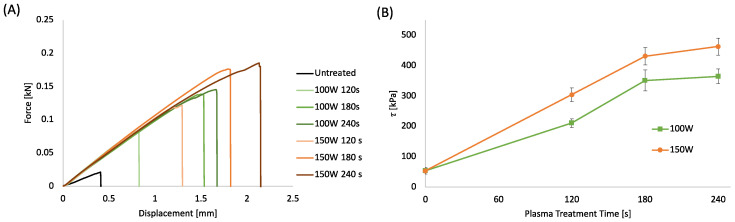
(**A**) Force-displacement curves of bonded PLA sheet 0.2 mm thick at different plasma treatment power and time; (**B**) Shear stress of bonded PLA sheets as a function of plasma treatment power and time.

In the frame of an environmentally sustainable approach for fabricating integrated WS without the use of solvents, glues, or adhesive tapes, plasma treatment was investigated to improve the bonding efficiency of the functional PLA layers. The bonding strength of the PLA sheets bonded via a hot laboratory press, following plasma treatment at different processing powers and times, was evaluated by shear stress analysis (Figure 3A,B). The plots in Figure 3A present the load as a function of the displacement. Figure 3B displays the shear stress at breaking point (*τ*) reported as a function of the plasma treatment time evaluated as the ratio between the maximum load and the contact surface. The load-displacement curves were characterized by a linear region suggesting the elastic behavior of the adhering interfaces, followed by a fragile fracture that always occurs at the interface [74]. The stiffness of the untreated samples is lower than the treated samples, suggesting that the plasma treatment induced a strong increase in surface adhesion. The plots in Figure 3B show that the shear stress of the samples increases almost linearly up to 180 s, reaching a maximum for the highest treatment time.

The increase in shear stress induced by the plasma treatment can be related to several factors, such as the increased surface energy that enables more intimate contact and ultimately enhances mechanical interlocking and interdiffusion of polymer chains between the surfaces [36]. Furthermore, the surface degradation due to the plasma etching at the interface can lead to a more pronounced interdiffusion of lower molecular weight polymer chains between the surfaces leading to a stronger bond [75]. Finally, the polar functional groups formed by the air-plasma treatment on the PLA surface can produce hydrogen or covalent bonds across the interphase capable of providing higher bond strengths [76].

**Figure 4:**
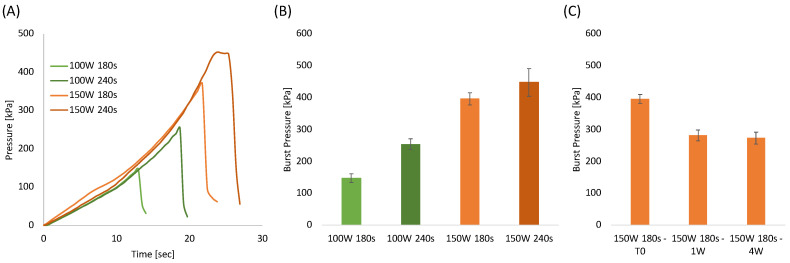
(**A**) Pressure-time curves of bonded PLA sheets at different plasma treatment power and time; (**B**) Burst pressure of bonded PLA sheets as a function of plasma treatment power and time; (**C**) Burst pressure of samples bonded at 150 W for 180 s as a function of aging time.

One of the most important requirements when bonding microstructured systems is the ability to seal channels so they can withstand high pressures efficiently. Therefore, burst test analysis was performed using the most optimized plasma treatment parameters as determined by the shear stress testing, i.e., plasma etching of 100 W and 150 W for a duration of 180 and 240 s, respectively. Figure 4A represents the pressure evaluated as a function of time with a constant water flow of 0.5 mL/h. The pressure curves clearly reveal that increasing the plasma duration and power increases robustness. Figure 4B, summarizes the burst pressure for each sample. In agreement with the shear stress tests, the best burst pressure values, around 400 kPa, and 450 kPa were obtained for the samples treated at 150 W for 180 and 240 s, respectively. 

The aging test conducted on samples stored in a closed polyethylene bag at ambient temperatures (Figure 4C) revealed that the burst pressure decreased after 1 week and that it remained constant after 4 weeks. This result was highly expected since it is well known that oxygenated groups formed on polymeric surfaces after plasma treatments are subjected to reorientation or migration of the induced polar chemical groups into the bulk of the polymer [73,77]. Morent et al. proved that air plasma-treated PLA surfaces lost around 56% and 70% of the treatment effect after 4 and 26 days, respectively [73]. In our case, the aging effect is significantly less than that observed by Morent et al. probably because the bonding in a hot press (performed within 3 h after plasma treatment) prevents most of the polymer chain mobility. Therefore, this limits the fraction of mobile groups which can reorientate or migrate from the PLA surface into the polymer bulk. From the results above, 150 W and 180 s were selected as the most adequate PLA etching conditions for the fabrication of the integrated sensors. 

### 3.3. Wearable Sweat Sensors Platform Design and Fabrication

**Figure 5:**
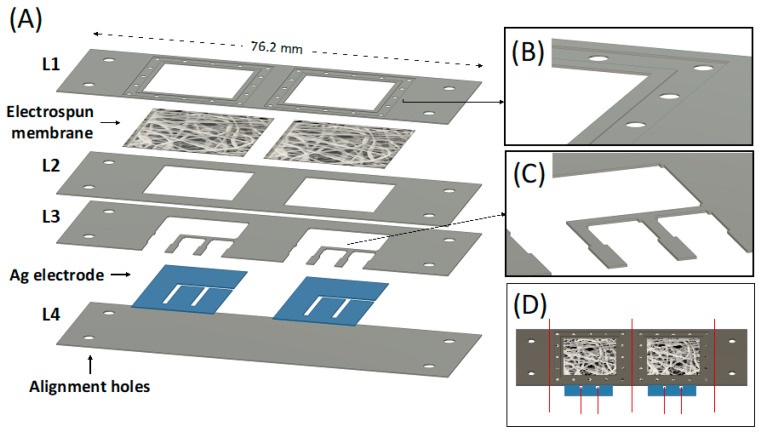
(**A**) CAD design of each individual PLA layer forming the whole device, including the electrospun membrane and the Ag electrode; (**B**) Detail of the membrane holder layer (L1) CAD design; (**C**) Detail of the upper electrode fixer layer (L3) CAD design; (**D**) CAD design of the whole device, the red line represents where to cut to obtain the final wearable device.

The PLA platform for WS was designed according to the scheme in Figure 5A. This design allows the fabrication of two integrated WS at the same time.

Four PLA layers of 76.2 mm × 25.4 mm with different thicknesses were used. The lower cover layer (L4) was 0.5 mm thick and acted as starting substrate, while all the other layers were maintained at 0.2 mm thick. The four holes present at the edge of each layer were used as an alignment system during the thermal bonding, coupling with the alignment pins integrated into the custom-made press plates. A membrane is integrated between the membrane holder layer (L1) and the membrane fixer layer (L2) to be in conformal contact with the skin and to drive the absorbed sweat directly on the top of the Ag electrodes via capillarity. The membrane holder layer (L1) was properly designed to hold the electrolytic solution of the electrolyte-assisted electrospinning processing and the deposited electrospun membrane. The square hole (side equal to 17.1 mm) was surrounded by a 1 mm wide engraved region (0.06 mm deep) surrounded by another engraved region (2 mm wide, 0.12 mm deep) obtainable via laser cutter by imposing a second and localized engraving process. The small holes (1 mm diameter) within this layer were designed to ease the electrolytic solution deposition for the electrolyte-assisted electrospinning processing. The L1 bottom side (magnified in Figure 5B) was designed to ensure adequate adhesion of the electrospun membrane after bonding with the membrane fixer layer (L2) and to avoid fluid leaking during the absorption. The design of the upper electrode fixer (L3) contains small pins (Figure 5C) to ensure the Ag electrodes’ adhesion to the lower cover layer (L4). The silver film was designed with the shape shown in Figure 5A and includes the working, counter, and reference electrodes. The as-cut silver film had a one-piece shape to ease its integration in the electrode aligner layers (L4). Figure 5D shows the design of the entire bonded chip. Finally, the sample is cut in correspondence with the red line (Figure 5D) to obtain the two individual complete sensors with 26.7 × 25.4 mm size.

**Figure 6:**
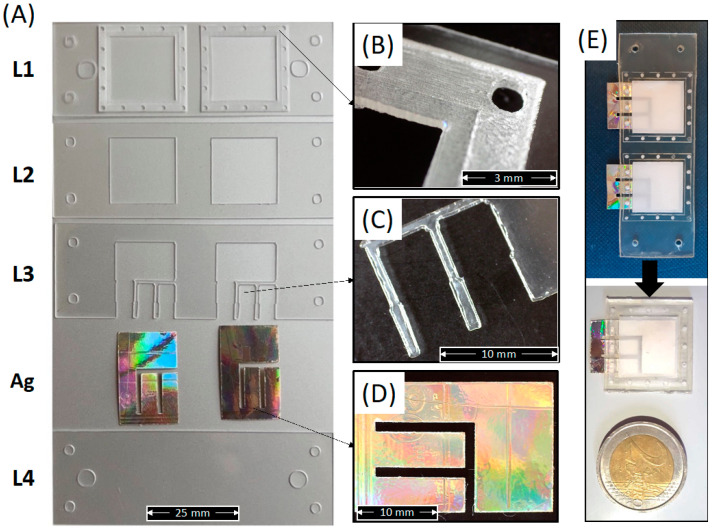
(**A**) Picture of each individual PLA layer forming the whole device, including the Ag electrodes; (**B**) Detail of the membrane holder layer (L1); (**C**) Detail of the upper electrode fixer layer (L3); (**D**) Detail of the Ag electrode; (**E**) Picture of the whole device after bonding and before or after cutting of the individual sensors.

Figure 6A shows the macroscopic shape of the as-cut individual PLA layers forming the whole device, including the Ag electrode. 

This figure clearly shows that the laser cutter allowed for obtaining well-defined and well-shaped sheets corresponding to the CAD design. The micrograph in Figure 6B shows a detailed image of the electrospun membrane holder layer obtained via a single (inner square) and double (outer square) engraving. Figure 6C shows a micrograph of the pins in L3 for the Ag electrode adhesion to the lower cover layer (L4). Figure 6D shows the one-piece shape of a single Ag electrode extracted from a compact disk and cut via laser cutter. Figure 6E show the picture of the whole integrated sensor before and after cutting into individual sensors according to the red lines in Figure 5D. The picture clearly reveals that the shape and the transparency of the PLA layers are also maintained after thermal bonding.

Figure 6 demonstrates the suitability of the processing route described above for the fabrication of miniaturized (weight of individual integrated sensor lower than 0.65 g), green, and wearable sensors made in PLA and integrating a PLA electrospun membrane as sweat sorption pad and Ag electrodes extracted from the compact disk.

### 3.4. PLA Electrospun Membrane Characterization

**Figure 7:**
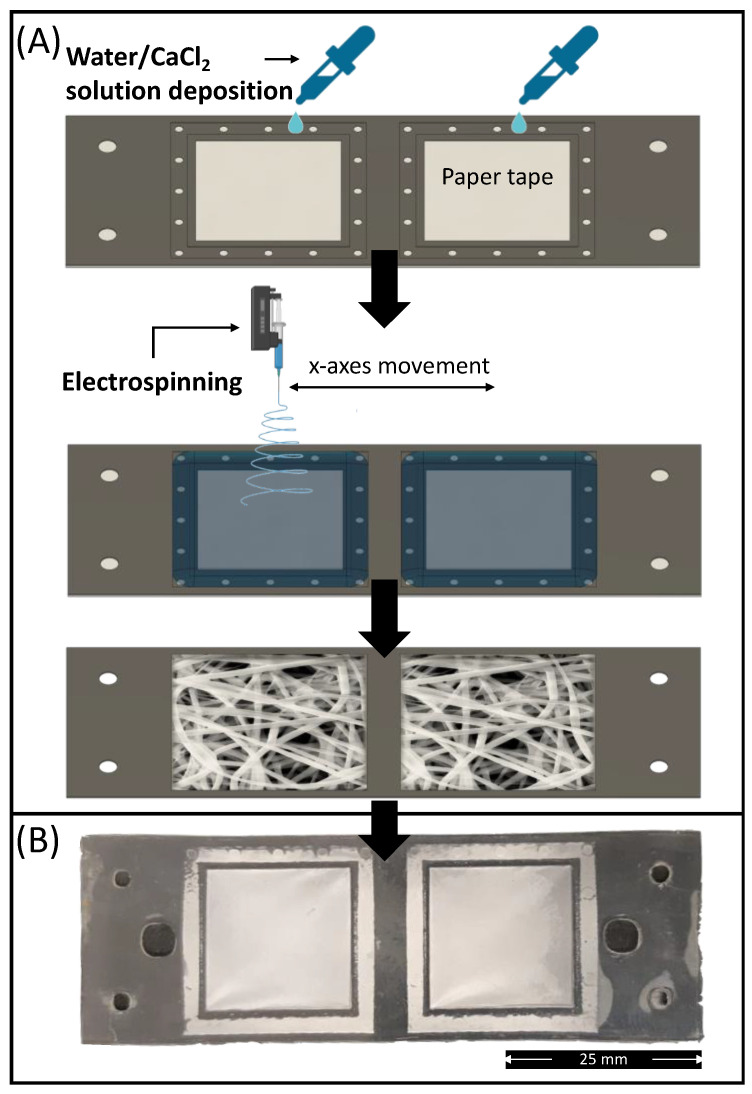
(**A**) Schematic representation of the electrospun PLA absorption PAD fabrication route via electrolyte-assisted electrospinning; (**B**) Picture of electrospun membrane deposited on the membrane holder layer (L1) bonded with L2.

The absorption pad unit of the device was composed of an electrospun PLA membrane prepared via electrolyte-assisted electrospinning directly on the membrane holder layer (L1). To prevent leaking of electrolyte solution (CaCl_2_/water at 10 wt%) used as static collector, the bottom of L1 (pre-processed via plasma treatment) was covered by means of a removable paper tape (Figure 7A).

After electrolytic solution deposition, the membrane holder sheet was positioned under the needle of the electrospinning equipment, and the membrane deposition was undertaken. After 5 min of processing, the electrospinning was stopped, and the electrolytic solution was removed from the collected samples together with the paper tape. The samples were subsequently washed in distilled water and dried for at least 2 days in a fume hood to remove any residual solvents. Finally, to improve the hydrophilicity of the electrospun membrane, the L1 layers containing the mats were processed under plasma treatment for 30 s at 50 W before bonding with the other layers. The electrolyte-assisted electrospinning approach provided a smooth and well-fitted membrane on the membrane holder shape (Figure 7B). 

**Figure 8:**
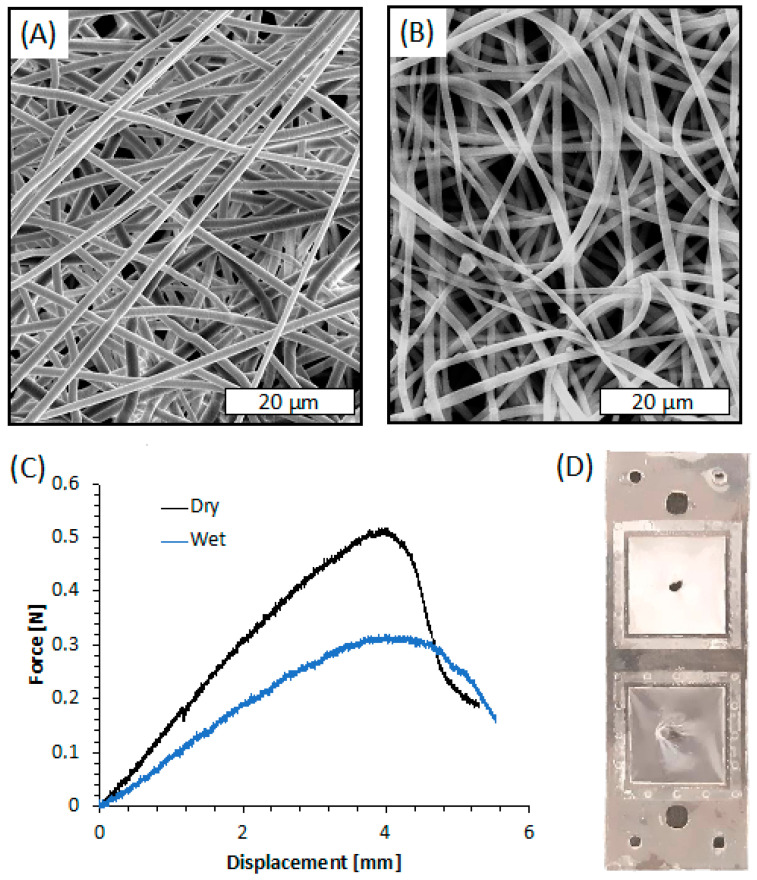
(**A**) SEM image of the electrospun membrane after plasma treatment; (**B**) SEM image of the electrospun membrane after plasma treatment and PLA layers’ bonding; (**C**) Force-displacement curves of the electrospun membrane obtained via indentation test; (**D**) Picture of the system after indentation test in dry-mode (upper square) and wet-mode (bottom square).

The SEM images of the membrane after plasma treatment, shown in Figure 8A, revealed smooth fibers in the microscale (mean diameter = 1.187 ± 0.261 µm). The fibers are randomly oriented and display a highly interconnected porous structure. The micrographs of the fibers shown in Figure 8A are comparable to those of the untreated samples, not shown for the sake of brevity. This result was highly expected since the plasma processing parameters were selected based on a previous work that demonstrated that these processing parameters did not affect the fiber morphology [38].

Since bonding of PLA sheets involves a duration in a hot press at a temperature near the T_g_ of PLA, SEM images of the electrospun membrane after its integration within the PLA layers were taken, as shown in Figure 8B. The image clearly reveals that the bonding procedure caused slight damage to the electrospun fibers closer to the press plate. They appear flatter than those observed before bonding, probably because of the temperature of the hot press, likely able to slightly modify the morphology of the thin PLA fiber although lower than Tg of PLA (61.5 °C, as reported in our previous work [78]). Nevertheless, the fiber diameter remained in the microscale (mean diameter = 1.312 ± 0.418 µm), and the interconnected porous structure was similar to that before bonding.

Indentation tests were carried out on the electrospun membrane deposited on L1 and bonded with L2 in order to evaluate its adhesion efficiency when integrated between the PLA layers. The representative force-displacement curves obtained by the indentation tests conducted on the PLA electrospun membranes, both dry and wetted with distilled water, are shown in Figure 8C. The curves of both dry and wet membranes are characterized by a linear elastic region followed by a rapid fracture. The stiffness of the membrane was evaluated as the ratio between the force and the displacement at the linear elastic region of the force-displacement curves. The dry samples exhibited higher stiffness (*k_dry_* = 0.14 ± 0.02 N/mm) and higher maximum load (*ML_dry_* = 0.51 ± 0.06 N) than wet systems (*k_wet_* = 0.09 ± 0.02 N/mm; *ML_wet_* = 0.32 ± 0.03 N). On the other hand, the maximum displacement of dry and wet membranes was comparable in the range of 3.8–4.3 mm. Interestingly, the fracture of the membranes always occurred at the indentation region, and there was no evidence of membrane slipping or layers delamination (Figure 8D).

These results indicated that the adhesion strength of the membranes on the PLA holder is higher than its mechanical resistance at the breakpoint both in dry and wet mode, thus confirming the suitability of the electrolyte-assisted electrospinning method for the fabrication of well-adhering and tight membranes.

To evaluate the suitability of the PLA membrane as a sweat absorption unit of the WS, the wettability and the water uptake of the electrospun membrane were investigated. The surface wettability of the membrane before and after plasma treatment was analyzed to evaluate their hydrophilic/hydrophobic characteristics through WCA measurements. As already observed in a previous work [38], electrospun PLA showed high intrinsic hydrophobicity, displaying a WCA value of around 131°. As expected, that value decreased to 0° following plasma treatment of the electrospun membrane [38]. The same hydrophilic character was observed in the samples integrated within the bonded PLA layers. The water uptake of the electrospun membranes revealed that the porous system could absorb up to 26 g/g of water. This value can be ascribed to the highly porous structure (porosity = 97%) and the low pore size (pore size~3 µm) of the electrospun membrane that can permit water absorption and retention via capillarity.

### 3.5. Electrochemical Chloride Detection

**Figure 9:**
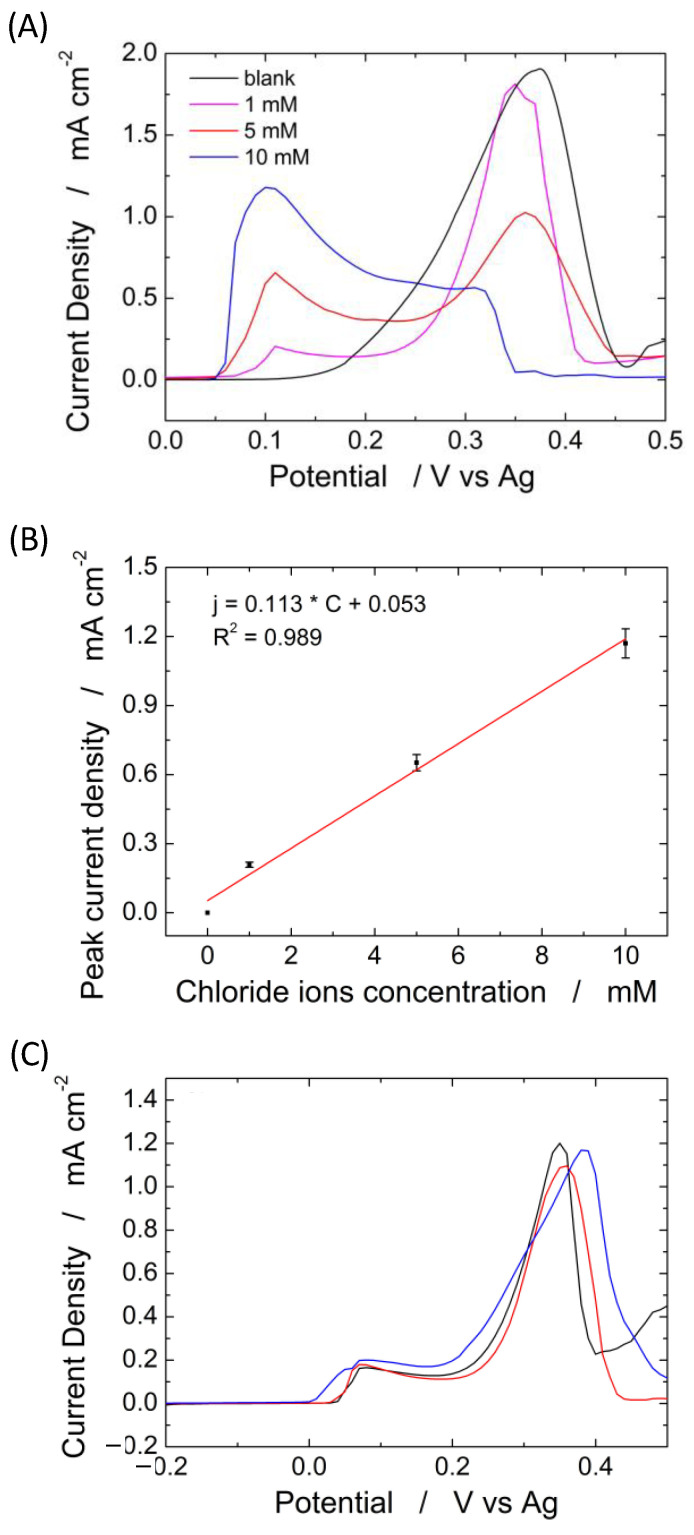
(**A**) Linear scan voltammetry obtained at different concentrations of chloride ions from 0 (black curve) to 10 mM and (**B**) corresponding calibration line; (**C**) linear scan voltammetry for sweat chloride quantification in a healthy volunteer using three different electrodes.

To establish the influence of the PLA membrane on the device operation of a sensor for Cl^−^ detection, different amounts of NaCl, ranging from 1 to 10 mM, were spiked on the electrode system with and without the PLA membrane. Chloride ions were detected by LSV, and the results are shown in Figure 9A–C. Figure 9A shows the LSV of the blank solution in the presence of a different amount of NaCl, using the device complete with an electrospun PLA membrane. In the absence of chloride ions (black curve), only one peak is observed at a potential of about 0.36 V vs. Ag, which is related to the oxidation of Ag to Ag^+^ [79]. When chloride ions are present in the solution, a new peak, located at 0.08 V, appears related to the oxidation of silver-to-silver chloride [80]. The peak related to the oxidation of Ag to Ag^+^ (0.36 V) decreases in amplitude with increasing concentration of chloride ions due to the formation of AgCl on the silver substrate that reduces the active sites available for silver oxidation. The amplitude of the peak at 0.08 V (oxidation of silver-to-silver chloride) linearly increases with chloride concentrations, as shown in Figure 9B. The slope of the linear regression corresponds to the sensor sensitivity [60]. Almost identical results were obtained in [66], where the same silver-based sensor was used for the identification of chloride ions but by immersing the sensor inside the solution. The LOD is 20 µm, and as demonstrated in [66], the sensors have excellent selectivity against many common interferents and also for the other halides.

The experiments in Figure 9 clearly show that the PLA membrane can be effectively used as an absorbent material, and its presence does not substantially modify a sensor’s electrochemical performance to quantify chloride. 

To verify the reproducibility, ten different devices were tested for the detection of 1 mM chloride using the system with and without the PLA membrane. The devices without PLA membrane exhibit a peak current density of 180 µA µM^−1^ cm^−2^ ± 5.5%, while the devices with PLA membrane exhibit a peak current density of 165 µA µM^−1^ cm^−2^ ± 4.8%. This is a very good result that shows both the reproducibility of the device (both in terms of fabrication and operation) and, again, the negligible interference of the PLA membrane on the operation of the device. The standard deviation of the proposed system is about 4.5% and possesses a very high selectivity, as demonstrated in our previous work [66].

To validate the proposed sensor platform, sweat chloride was quantified prior to dilution of the sweat collected from a healthy volunteer with a standard solution of 0.1 M sodium nitrate (diluting factor = 30), Figure 9C. The test was performed in triplicate, and the results showed a concentration of Cl^−^ of 32.29 ± 3.8 mM. This value is in agreement with the standard concentration of chloride ions in human sweat [81]. The Cl^−^ concentration in the real sweat was also measured by the standard titration method. The measured value is 32.94 ± 5 mM confirming the good performance of the sensor. 

The results obtained clearly show the ability of the proposed sensor platform to quantify sweat chloride with very high reproducibility and low sample volume in real time and even directly on the skin, thanks to the PLA absorbent membrane.

**Figure 10:**
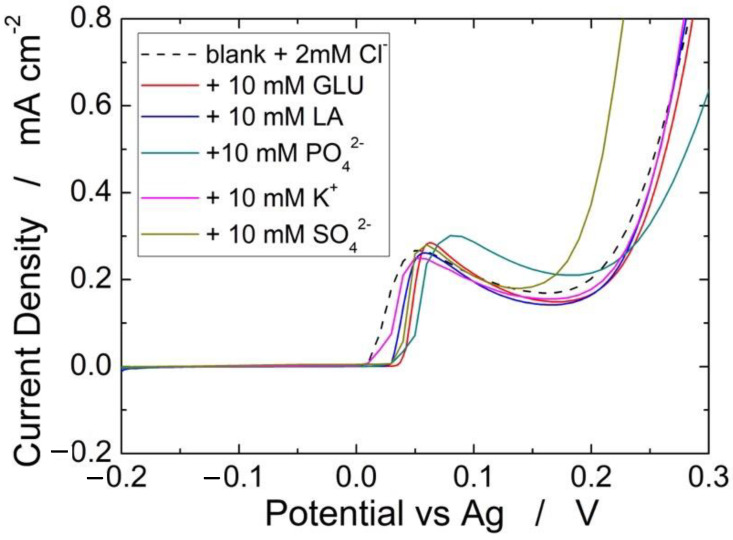
Effect of different interferent species on the detection of 2 mM Cl^−^.

Figure 10 shows the interference of different species (glucose, lactic acid, PO_4_^2−^, K+, and SO_4_^2−^) on the detection of 2 mM of Cl^−^. A very negligible interference, both in terms of peak potential and intensity, was found for all investigated species. For each species, the interference test was repeated three times, finding a very high reproducibility because in all cases we measured variations of less than 5%. Similar results were obtained in in [66], where the same Ag-based sensor was full characterized but without PLA absorbent membrane. Thus, it is possible to assert that the proposed sensor has a very excellent selectivity and reproducibility.

## 4. Conclusions

In this work, a novel and environmentally sustainable route for the fabrication of wearable sweat sensors is proposed. The flat-die extrusion processing of PLA allowed for obtaining transparent sheets with modulable thicknesses ranging from 0.2 to 0.5 mm. The processing conditions of an air plasma treatment, coupled with thermal bonding, were optimized to ensure high adhesion strength and transparency on PLA sheets without affecting the micropatterns of the device. The rapid prototyping of the individual PLA layers composing the final sensor was carried out by means of a laser cutter apparatus according to a CAD design. Laser cutter parameters were optimized to obtain well-shaped micrometric functional features on the PLA extruded sheets. 

An electrolyte-assisted electrospinning procedure was followed for the direct deposition of microfibrous PLA mats on the membrane holder layer resulting in smooth and well-fitted. The morphology of the electrospun membranes revealed fibers in the microscale that were slightly damaged by the bonding procedure without compromising their functionality. The membrane holder design allowed obtaining membrane adhesion strength between the bonded PLA sheets higher than its mechanical resistance at the break. The wettability of the highly porous membrane was improved via air-plasma pre-treatment to ensure rapid sweat absorption allowing the sensor to be directly applied to the skin.

To obtain the final device, the PLA sheets were bonded with both PLA membrane and silver-based electrodes obtained from a compact disc. The obtained device was used as an electrochemical sensor for detecting Cl^−^ ions in the sweat. Prior to sweat analysis, the sensor was calibrated in the range from 1 to 10 mM using different concentrations of NaCl in water-based samples. The device was tested with and without PLA membrane, obtaining almost identical results. The results showed that the bonding procedure and the presence of PLA membrane did not affect the sensor performance in terms of sensitivity, selectivity and signal read-out.

To validate the sensor real sweat sample was collected from a healthy volunteer. The same sweat sample was tested with both our device and the standard titration method obtaining comparable results. Chloride ions measurements in real sweat agreed with known average values, thus demonstrating the potential of the sensor as a sustainable point-of-care device.

## Data Availability

The data presented in this study are available on request from the corresponding author.
